# Upconversion Nanoparticle-Based Förster Resonance Energy Transfer for Detecting DNA Methylation

**DOI:** 10.3390/s16081259

**Published:** 2016-08-09

**Authors:** Seockjune Kim, Sang-Hyun Hwang, Su-Gyeong Im, Min-Ki Lee, Chang-Hun Lee, Sang Jun Son, Heung-Bum Oh

**Affiliations:** 1 Department of Laboratory Medicine, University of Ulsan College of Medicine and Asan Medical Center, Seoul 05505, Korea; sj1kim1@gmail.com; 2 Department of Laboratory Medicine, Center for Diagnostic Oncology, Research Institute and Hospital, National Cancer Center, Goyang-si 10408, Korea; relationship01@ncc.re.kr; 3 Hematologic Malignancy Branch, Research Institute and Hospital, National Cancer Center, Goyang-si 10408, Korea; 4 Department of Internal Medicine, Pusan National University School of Medicine and Biomedical Research Institute, Pusan National University Hospital, 179 Gudeok-ro, Seo-gu, Busan 602-739, Korea; leemk@pusan.ac.kr; 5 Department of Pathology, Pusan National University School of Medicine and Biomedical Research Institute, Pusan National University Hospital, 179 Gudeok-ro, Seo-gu, Busan 602-739, Korea; cnlee@pusan.ac.kr; 6 Department of Chemistry, Gachon University, Seongnam, Gyeonggi, and Gachon Medical Research Institute, Gil Medical Center, Inchon 461-701, Korea; triaza@gmail.com

**Keywords:** DNA methylation, *CDKN2A*, upconversion nanoparticle, FRET, biosensing, MSP, pyrosequencing, MS-UC-FRET

## Abstract

Aberrant methylation of a crucial CpG island is the main mechanism for the inactivation of *CDKN2A* in the early stages of carcinogenesis. Therefore, the detection of DNA methylation with high sensitivity and specificity is important, and various detection methods have been developed. Recently, upconversion nanoparticles (UCNPs) have been found to display a high signal-to-noise ratio and no photobleaching, making them useful for diagnostic applications. In this pilot study, we applied UCNPs to the detection of *CDKN2A* methylation and evaluated the feasibility of this system for use in molecular diagnostics. DNA PCR was performed using biotinylated primers, and the PCR amplicon was then intercalated with SYTOX Orange dye, followed by incubation with streptavidin-conjugated UCNPs. Fluorescence detection of the Förster resonance energy transfer (FRET) of the UCNPs (MS-UC-FRET) was then performed, and the results were compared to those from real-time PCR (RQ-PCR) and pyrosequencing. Detection by MS-UC-FRET was more sensitive than that by either RQ-PCR or pyrosequencing. Our results confirmed the success of our MS-UC-FRET system for detecting DNA methylation and demonstrated the potential application of this system in molecular diagnostics.

## 1. Introduction

Gene silencing by aberrant promoter hypermethylation is now recognized as a crucial component in cancer initiation and progression [1]. For example, alterations to *CDKN2A*, a tumor suppressor gene that encodes a specific inhibitor of cyclin-dependent kinase (CDK) 4 and 6, are found in a wide range of human cancers [2]. Frequent, aberrant methylation of a crucial CpG island was found to be the main mechanism for inactivation of *CDKN2A* in the early stages of carcinogenesis [3], and the degree of *CDKN2A* methylation has been associated with overall survival and disease-free survival in patients with non-small cell lung cancer (NSCLC) [4]. Therefore, the assay and detection of DNA methylation with high sensitivity and specificity is important for the diagnosis and prognosis of patients with NSCLC associated with aberrant DNA methylation [5]. To date, various detection methods have been developed for determining the aberrant methylation status of primary tumors [6], and PCR-based methods that use sodium bisulfite-treated DNA as a template are generally accepted as the most analytically-sensitive and specific techniques for analyzing DNA methylation at a single locus [7]. Methylation-specific PCR (MSP) [8], MethyLight [9] and pyrosequencing [10] are the traditional PCR-based methods widely used for analyzing DNA methylation at a single locus. 

Recently, new strategies have been proposed for the optical detection of DNA methylation levels by fluorescence resonance energy transfer (FRET) [11] and label-free fluorescence [12]. Because of their high sensitivity and specificity, fluorescence-based methods have attracted much attention for use in DNA sequence detection [13]. Traditional fluorophores, like fluorescent dyes, are based mainly on downconversion fluorescence, which has several drawbacks, including photobleaching and high background noise from autofluorescence [14]. 

Recently, there has been increasing interest in developing upconversion nanoparticles (UCNPs) that can emit higher energy, visible photons after absorbing low-energy, near-infrared photons (infrared light, 980 nm) [15]. Compared to traditional downconversion fluorophores, UCNPs have unique optical and chemical properties, such as a large Stokes shift, good photostability and a high light-penetration depth [16]. Therefore, UCNPs are attractive due to their unique physical and electrochemical properties, which have made UCNP-based techniques useful for detecting nucleic acids with high sensitivity, including microRNA [17], hepatitis B viral DNA [18], a T790M mutation in the epidermal growth factor receptor [19] and the *Mycobacterium tuberculosis* complex [20]. 

In this study, we evaluated the feasibility of detecting DNA methylation using a UCNP-based FRET technique. Similar to our previous study [20], we amplified methylated DNA by PCR and detected methylation using FRET between UCNPs and intercalating SYTOX Orange dye. 

## 2. Experimental Section

### 2.1. Tissue Specimens

Tissue collection and analyses in this study were approved by the institutional review board of the Pusan National University Hospital (IRB No. 2012-3). A total of 49 DNA samples (23 adenocarcinoma, 26 squamous cell carcinoma), extracted from NSCLC tissues of patients with NSCLC, were provided by the Pusan National University Hospital, a member of the National Biobank of Korea, which is supported by the Ministry of Health, Welfare and Family Affairs. All samples derived from the National Biobank of Korea were obtained with informed consent under institutional review board-approved protocols. 

The QIAamp DNA Mini Kit and the QIAcube (both from Qiagen, Hilden, Germany) were used to extract DNA from tissue samples according to the manufacturer’s instructions. 

### 2.2. Bisulfite Treatment

Eluted DNA (20 µL) was modified using the EZ DNA Methylation-Lightning Kit (Zymo Research, Orange, CA, USA) according to the manufacturer’s protocol. Final elution of the DNA was performed in 20 µL of Tris-EDTA buffer (10 mmol/L Tris, 1 mmol/L EDTA, pH 8.0). Bisulfite-treated DNA was used as the target for investigating the hypermethylation status of the *CDKN2A* promoter as described previously [21]. 

### 2.3. Detection of CDKN2A Methylation by Methylation-Specific PCR

The previously-described primers were used for methylation-specific PCR (MSP) analysis [8]. The forward primer, 5′-biotin-TTATTAGAGGGTGGGGCGGATCGC-3′, and the reverse primer, 5′-GACCCCGAACCGCGACCGTAA-3′, were used to amplify the methylated *CDKN2A* promoter. The HotStarTaq Plus Master Mix Kit (Qiagen) was used for the PCR amplification, where each reaction contained dNTPs, each primer at a final concentration of 100 nM and 5 µL of extracted DNA. A GeneAmp 9700 thermal cycler (Applied Biosystems, Foster City, CA, USA) was used for the DNA amplification. For the PCR, the conditions were as follows: 10 min at 95 °C; followed by 40 cycles at 94 °C for 30 s, 65 °C for 45 s and 72 °C for 30 s; and a final step at 72 °C for 10 min. In all PCR reactions, universal methylated and unmethylated DNA (Qiagen, Hilden, Germany) were used as positive and negative controls, respectively. The PCR products were analyzed by electrophoresis on a 2% agarose gel. 

### 2.4. Preparation of UCNPs

UCNPs were prepared as previously reported [20,22]. All chemicals were purchased from Sigma-Aldrich (St. Louis, MO, USA). The UCNP synthesis, silica coating and (3-aminopropyl) triethoxysilane (APTES) modifications were as previously described [22]. 

### 2.5. Detection of CDKN2A Methylation by Methylation-Specific UCNP-FRET

**Figure 1:**
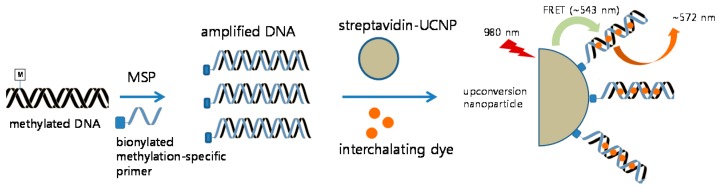
Schematic procedures for the detection of methylated DNA based on Förster resonance energy transfer (FRET) using upconversion nanoparticles (UCNPs) and intercalating dye. Briefly, bisulfite-converted DNA was amplified using methylation-specific PCR, in which the forward primer was biotinylated. Streptavidin-UCNPs and SYTOX Orange were mixed with the PCR product. Consequently, the methylated PCR products were detected using FRET between UCNPs and intercalating SYTOX Orange dye.

Primers selected for binding to UCNPs were the same sequence as those of MSP, and the forward primer was biotin-labeled at the 5’ end of the primer (Integrated DNA Technologies (IDT), Coralville, IA, USA). Reaction conditions were set as reported previously [20]. Briefly, extracted DNA was treated with bisulfite conversion, wherein methylated cytosines remained unaffected. Bisulfite-converted DNA was amplified using methylation-specific PCR, in which the forward primer was biotinylated. Streptavidin-UCNPs (1 µL) were mixed with 2 µL of the PCR product, and the mixture was left undisturbed for 15 min. Then, 1 µL of 12.5 µM SYTOX Orange (Invitrogen, Carlsbad, CA, USA) was added. Consequently, the methylated PCR products were detected by fluorescence emission using FRET between UCNPs and intercalating SYTOX Orange dye as previously described (Figure 1) [20]. Fluorescence profiles of the UCNPs and SYTOX Orange were measured at 543 and 572 nm, respectively. The FRET ratio_sample_ refers to the ratio of the emission intensities of SYTOX Orange to UCNP (F_572 nm_/F_543 nm_) for the sample, whereas the FRET ratio_control_ serves as the background and refers to the FRET ratio for the negative non-template control. 

### 2.6. Detection of CDKN2A Methylation by Real-Time PCR 

For comparison with UCNP-based methylation detection, RQ-PCR with primers and probes to detect methylation of *CDKN2A* and *ACTB* was performed as previously described [23,24]. Briefly, for p16, the forward primer (5′-TGGAGTTTTCGGTTGATTGGTT), reverse primer (5′-AACAACGCCCGCACCTCCT) and probe (FAM5′-ACCCGACCCCGAACCGCG-BHQ-1) combination were used. The reference gene, *ACTB*, was also amplified with the forward primer (5′-TGGTGATGGAGGAGGTTTAGTAAGT), reverse primer (5′-AACCAATAAAACCTACTCCTCCCTTAA), and probe (FAM5′-ACCACCACCCAACACACAATAACAAACACA-3′TAMRA). TaqMan Universal Master Mix II (Qiagen) was used for the PCR amplification. DNA was amplified using an ABI 7300 instrument (Applied Biosystems, Foster City, CA, USA) under the following conditions: 10 min at 95 °C; followed by 45 cycles at 95 °C for 15 s and 60 °C for 60 s. The average Ct value of duplicates was used in calculations. 

### 2.7. Detection of CDKN2A Methylation by Pyrosequencing

Forward primer, 5′-TGGTTTTTGTTATAAAAATTATGATTGTAA, reverse primer, 5′-AATAATTACCCAAAACCCCAATAATAAC-3′-biotin, and the sequencing primer, 5′-AAATTATGATTGTAAAATAT, were used for pyrosequencing according to the procedure as previously described [21,25]. Pyrosequencing was performed using a PSQ96MA system (Biotage, Uppsala, Sweden) according to the manufacturer’s protocol. The assay was validated by an internal control (a non-CpG cytosine in the targeted methylation sequence region). Methylation of p16 for each sample was calculated as the average value of five CpGs (mC/total C × 100 (%)) of the promoter CpG islands examined.

### 2.8. Detection Sensitivity of MS-UC-FRET

We compared the detection rates of methylation detection techniques (MS-UC-FRET, pyrosequencing and RQ-PCR) using serial samples of methylated bisulfite-converted DNA mixtures. We prepared methylated bisulfite-converted DNA mixtures (with a methylation percentage of 50%, 20%, 10%, 5%, 1%, 0.1%, 0.05% and 0.01%) with unmethylated bisulfite-converted DNA (Qiagen) and fully-methylated DNA (Qiagen). 

### 2.9. Detection of Methylated DNA in Lung Cancer Tissues

We used 49 tissue samples for detecting methylated *CDKN2A* DNA in patients with lung cancer. Results from the MS-UC-FRET technique were compared to those obtained by RQ-PCR and pyrosequencing (MSP as a reference method in this study). Receiver-operating-characteristics (ROC) curve analysis was performed to evaluate the diagnostic performance of these techniques.

## 3. Results

**Figure 2:**
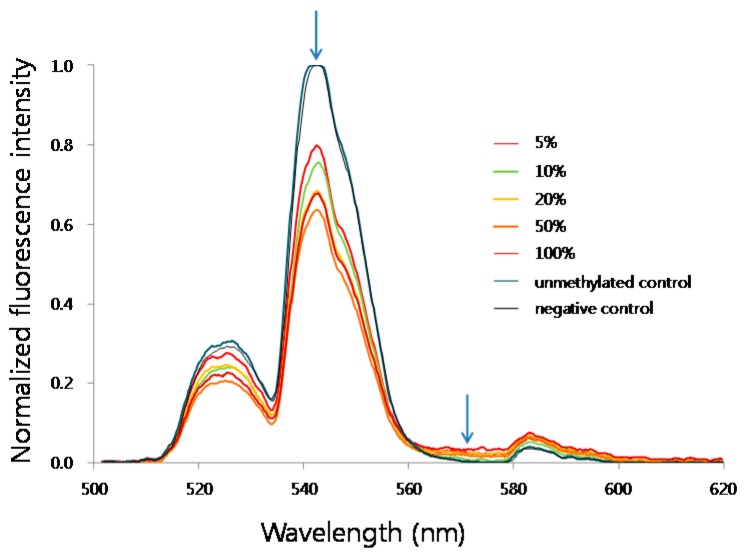
Representative fluorescence spectra of MS-UC-FRET output for methylated samples of serial methylation percentages ranged from 0% (unmethylated control DNA) to 100% (fully methylated control DNA). Arrows indicate the excitation (emission peak of UCNP) and emission peaks of SYTOX Orange, respectively.

The principle of this design is shown in Figure 1. The biotinylated, methylated amplicon was hybridized to streptavidin-UCNPs. After intercalation of SYTOX Orange to the streptavidin-UCNPs, FRET between the UCNPs and SYTOX Orange occurred, and the intensity of the green fluorescence (at 543 nm) from the UCNPs decreased. Figure 2 shows representative fluorescence spectra of the MS-UC-FRET output. 

### 3.1. Detection Sensitivity of MS-UC-FRET

**Figure 3:**
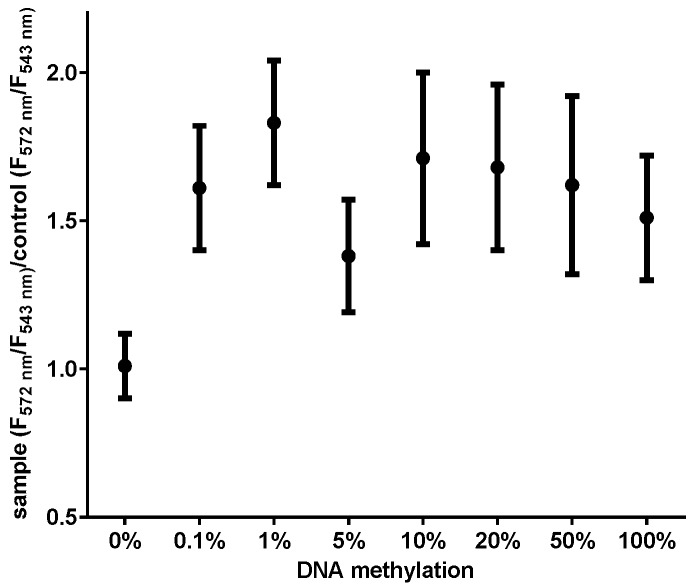
Serial dilution testing for the detection of methylated DNA. The values of F_572_/F_543_ were used for the detection of *CDKN2A* DNA, where F_543_ is the fluorescence of upconversion nanoparticles (donor) at 543 nm and F_572_ is the fluorescence of SYTOX Orange (acceptor) at 572 nm during FRET. To normalize the variation of each measurement, the ratio of sample (F_572_/F_543_)/control (F_572_/F_543_) was used. The control was a mixture of UCNPs, SYTOX Orange and PCR master mix without DNA template. The methylation of *CDKN2A* varied from 0.01% to 100%. Error bars represent the standard deviations of repeated measurements.

We prepared a range of methylated DNA mixtures (0.01%–100%) to study the detection sensitivity of the MS-UC-FRET method. The mixtures were assayed in replicates (n = 10) every day for five days to capture the variability and reproducibility of the detection technology. FRET signals were consistently observed for as little as 0.1% of methylated DNA (ten reactions had *CDKN2A* detected 100% of the time; Figure 3). The estimated lowest detection of *CDKN2A* methylation for real-time PCR and pyrosequencing was 1% and 5%, respectively (Table 1). Therefore, the methylation detection of the MS-UC-FRET method was more sensitive than RQ-PCR and pyrosequencing.

### 3.2. CDKN2A Methylation in Lung Cancer Tissue by MS-UC-FRET

**Figure 4:**
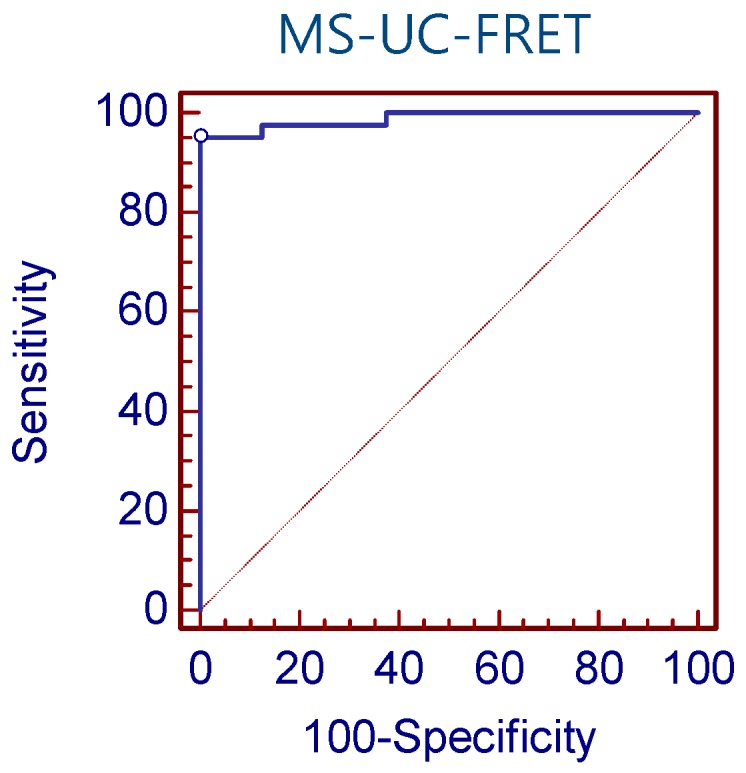
ROC curve analysis of MS-UC-FRET. According to ROC curve analysis, the sensitivity and specificity of MS-UC-FRET (a cut-off value of 1.11) were 95.1% and 100.0%, respectively.

We compared the techniques for the detection of *CDKN2A* methylation in NSCLC tissues (n = 49; Table S1). According to ROC curve analysis, the sensitivity and specificity of MS-UC-FRET (a cut-off value of 1.11) were 95.1% and 100.0%, respectively (Figure 4). Using a cut-off value of 5.42% of pyrosequencing, the sensitivity was 43.9%, and the specificity was 100.0%. The sensitivity and specificity of RQ-PCR was 36.6% and 100.0%, respectively. The detection rate of methylation by MS-UC-FRET (79.6%, 39/49) was higher than both pyrosequencing (36.7%, 18/49) and RQ-PCR (30.6%, 15/49).

## 4. Discussion

In this study, we used UCNPs as FRET donors to detect DNA methylation based on UCNP intercalating dye-based FRET, which had been previously applied to detect the *IS6110* sequence of the *Mycobacterium tuberculosis* complex [20]. Methylated DNA PCR amplicons were mixed with streptavidin-conjugated UCNPs. Through intercalation with SYTOX Orange dye, FRET occurred between UCNPs and the intercalating dye [26]. The methylated-*CDKN2A* positive rate and the diagnostic performance of the UC-MS-FRET method were higher than those of RQ-PCR and pyrosequencing.

The majority of methods used to detect gene-specific methylation include MSP, nested MSP and RQ-PCR, which uses sodium bisulfite-treated DNA as a template [7]. Traditional MSP is a sensitive methylation detection method, but its limitations include the risk of false positives and its qualitative nature. Quantitative MS-PCR (qMS-PCR), which uses fluorescent hydrolysis probes, overcomes most of the problems associated with traditional MSP [7]. RQ-PCR offers a quantification method, but is limited by inherent background fluorescence, which makes correct background removal important [11]. Bisulfite sequencing is widely used for mapping promoter hypermethylation and epigenotyping [27].

Recently, a series of nanoparticles, including quantum dots (QDs), UCNPs and gold nanoparticles (AuNPs), has been used in FRET assays for biosensing DNA methylation and has exhibited higher sensitivities and better stabilities compared to traditional organic fluorophores [26]. Bailey et al. reported a highly sensitive and quantitative methylation-specific QD-FRET assay for the detection of methylation status, and the key features of MS-qFRET using QD include its low intrinsic background noise and high resolution [11]. 

Here, we have designed UCNP-FRET-based DNA methylation assays and show that UCNP-FRET can distinguish as low as 0.1% methylation in *CDKN2A* from DNA mixtures. 

The *CDKN2A* methylation rate was determined to be 79.6% in NSCLC tissues by MS-UC-FRET, 30.6% by RQ-PCR and 36.7%% by pyrosequencing in this study. Abnormal *CDKN2A* promoter hypermethylation has been found in several types of tumor, and the frequency of *CDKN2A* promoter methylation ranged from 17% to 80% (median 44%) in lung cancer tissues [28]. Hypermethylated *CDKN2A* promoter sequences were observed in 80.2% of tumor tissues by MSP [29], as a function of several patient-, disease- or technical-related factors [30]. MSP is known for its high analytical sensitivity and has been reported to detect the 0.1% methylated template in an excess of unmethylated DNA [8]. In RQ-PCR-based detection, *CDKN2A* was found to be hypermethylated in 29% of NSCLC tissues [31] and in 30.2% by pyrosequencing [32]. 

In this study, we developed an MS-UC-FRET system for the detection of *CDKN2A* methylation and demonstrated the potential application of this system in molecular diagnostics. In comparison with RQ-PCR and pyrosequencing-based methylation detection, our MS-UC-FRET system was more sensitive. However, there were some limitations in this study. The FRET efficiency in the design of MS-UC-FRET at this proof of concept stage was still low, and the variation at each batch was relatively high. Thus, further studies should be done to improve this method by controlling the size and shape of UCNPs or choosing other nanomaterials, such as graphene oxide, QD or AuNP, therefore enhancing the FRET efficiency. The analytical sensitivity of MS-UC-FRET is also influenced by primer design, the number of PCR cycles and annealing temperature, similar to MSP methods. 

Many PCR-based nucleic acid detection methods using nanoparticles have been reported, and the paradigm for current molecular diagnostic methods is shifting from qualitative PCR to a rapid, quantitative and homogeneous method with no separation steps, such as quantitative RQ-PCR [33] and nanoparticle-based FRET methods [7]. Based on UCNPs displaying a high signal-to-noise ratio and no photobleaching, their use as DNA sensors has demonstrated high sensitivity and specificity [34]. 

In conclusion, we have developed a new UCNP-based FRET system for the detection of *CDKN2A* methylation, where a limit of detection as low as 0.1% was achieved. In this study, we showed that the performance of a UCNP-based detection system was more sensitive than RQ-PCR and pyrosequencing. 

## Figures and Tables

**Table 1:** Detection sensitivity of MS-UC-FRET, RQ-PCR and pyrosequencing for *CDKN2A* methylation (number of replicates with *CDKN2A* methylation detected).

**Methylation%**	0	0.01	0.05	0.1	1	5	10	20	50	100
	No. positive/No. tested									
UC-MS-FRET	NEG	4/10	2/10	10/10	10/10	10/10	10/10	10/10	10/10	10/10
RQ-PCR	0/4	0/4	0/4	0/4	0/4	4/4	4/4	4/4	4/4	4/4
pyrosequencing	NEG	ND	ND	ND	ND	2/2	2/2	2/2	2/2	ND

Abbreviations: NEG: negative; ND: not done.

## Supplementary Materials

The following is available online at http://www.mdpi.com/1424-8220/16/8/1259/s1, Table S1: Detection of methylation in lung cancer tissue samples (n = 49).
