# Supplementary material for: Upconversion Nanoparticle-Based Förster Resonance Energy Transfer for Detecting DNA Methylation
Source: Sensors (Basel). 2016 Aug 9;16(8):1259. doi: 10.3390/s16081259 (PMC5017424; doi:10.3390/s16081259)
Supplement: Supplementary file 1 [file sensors-16-01259-s001.pdf]

Supplementary Materials: Upconversion Nanoparticle-Based Förster Resonance Energy Transfer for Detecting DNA Methylation

Seockjune Kim, Sang-Hyun Hwang, Su-Gyeong Im, Min-Ki Lee, Chang-Hun Lee, Sang Jun Son and Heung-Bum Oh

Table S1. Detection of methylation in lung cancer tissue samples (n = 49).

Sample No.	MSP	MS-UC-FRET Ratio	RQ-PCR (C_T)	Pyrosequencing (%)
1	negative	0.97	undected	4.1
2	positive	1.24	undected	5.1
3	positive	1.49	31.8	6.5
4	positive	1.21	32.4	5.5
5	negative	0.92	undected	3.0
6	positive	1.31	34.9	5.6
7	positive	1.54	38.1	4.5
8	positive	1.10	undected	3.9
9	negative	0.88	undected	3.7
10	positive	1.43	29.9	9.1
11	positive	1.64	26.9	39.0
12	negative	0.92	undected	2.8
13	positive	1.15	undected	3.0
14	positive	1.47	undected	3.7
15	negative	1.00	undected	2.6
16	positive	1.16	undected	2.6
17	positive	1.41	undected	3.4
18	positive	1.52	27.5	60.5
19	negative	1.06	undected	2.1
20	negative	1.06	undected	3.9
21	positive	1.54	28.0	36.1
22	positive	1.43	undected	4.3
23	positive	1.46	undected	3.1
24	positive	1.61	28.0	47.6
25	positive	1.28	undected	4.1
26	positive	1.50	undected	5.0
27	positive	1.36	undected	4.0
28	positive	1.47	undected	5.1
29	positive	1.46	27.0	77.8
30	positive	1.34	undected	3.9
31	positive	1.76	28.1	33.8
32	positive	1.40	undected	7.9
33	positive	1.79	29.0	36.0
34	positive	1.49	39.6	5.0
35	positive	1.65	undected	3.9
36	positive	1.62	undected	3.9
37	positive	2.18	28.0	47.74

Table S1. *Cont.*

Sample No.	MSP	MS-UC-FRET Ratio	RQ-PCR (C_T)	Pyrosequencing (%)
38	positive	1.33	undected	3.22
39	positive	1.29	undected	2.96
40	positive	1.43	undected	5.21
41	positive	1.23	undected	4.13
42	positive	1.38	undected	5.56
43	positive	1.45	undected	5.26
44	negative	1.11	undected	5.42
45	positive	2.09	27.8	55.79
46	positive	1.02	undected	5.71
47	positive	1.26	undected	5.15
48	positive	1.34	undected	6.29
49	positive	1.16	undected	6.63
